# Maxillary expansion or contraction and occlusal contact adjustment: effectiveness of current aligner treatment

**DOI:** 10.1007/s00784-021-03780-4

**Published:** 2021-01-20

**Authors:** Ulrike Riede, Sandra Wai, Sabrina Neururer, Bärbel Reistenhofer, Gregor Riede, Katharina Besser, Adriano Crismani

**Affiliations:** 1 Department of Orthodontics, Medical University of Innsbruck, Innsbruck, Austria; 2 Department of Medical Statistics, Informatics and Health Economics, Medical University of Innsbruck, Innsbruck, Austria; 3 Private Practice of Orthodontics, Vienna, Austria; 4 Private Practice of Oral and Maxillofacial Surgery, Jenbach, Austria

**Keywords:** Aligner treatment, Invisalign®, Maxillary expansion, Maxillary contraction, Occlusal contacts, SmartTrack®

## Abstract

**Objectives:**

To evaluate the precision of aligner (Invisalign®) treatment with the current material (SmartTrack®) in achieving expansion or contraction of the maxilla and occlusal contacts as simulated in the proprietary planning software (ClinCheck®, CC).

**Materials and methods:**

Thirty patients thus treated were retrospectively evaluated. Four maxillary models were analyzed per patient: a pretreatment model, a scan-based CC model, a posttreatment clinical model, and a CC model reflecting the treatment outcome as initially simulated. Thirteen transverse parameters were measured on each model separately by two investigators. Occlusal contacts were also analyzed.

**Results:**

The measuring method was validated by both investigators arriving at similar results for the effectiveness by which the simulated treatment goals had been clinically achieved. Significant differences (*p* < 0.05; Wilcoxon signed-rank test) were observed for transfer precision from the casts to the planning software and between the simulated and clinical outcomes. Intense occlusal contacts in the simulations materialized less common (≈ 2%) than ideal contacts (≈ 60%) in the clinical outcomes.

**Conclusions:**

The effectiveness of achieving the simulated transverse goals was 45% and was generally not found to be better with SmartTrack® than with the previously used Ex30® material. Out of 100 simulated occlusal contacts, 40 will never materialize, and achieving around 60 will adequately ensure a clinically favorable contact pattern.

**Clinical relevance:**

With the caveat that any overcorrection will to some extent reduce the precision, it seems perfectly possible to make deliberate use of overcorrection in current aligner therapies for transverse maxillary expansion or contraction.

## Introduction

Being one of the most advanced modalities of orthodontic treatment, the Invisalign® (Align Technology, Santa Clara, CA, USA) concept offers benefits for patients and clinicians. These aligners, given the deceptive simplicity by which they correct malocclusion, are used not only by orthodontists but by many dental practitioners. They entail less of an esthetic compromise than a fixed buccal multibracket appliance and fewer functional limitations than a lingual appliance in adult patients [1]. As critical advantages over multibracket appliances, they also involve a lower incidence of demineralization, enamel abrasion, periodontal lesions, and mucosal irritations [2, 3].

What is clinically achieved by the end of aligner treatment should match the therapeutic goal that was previously simulated by modifying the patient’s initial tooth setup in the planning software (ClinCheck®; Align Technology). Djeu et al. [4] reported less precision of Invisalign® treatment compared to conventional multibracket appliances. Simon et al. [5] and Kravitz et al. [6] assessed in a retrospective and prospective study discrepancies between the previously simulated goals and the clinical outcomes of aligner treatment with a view to considering overcorrection of specific tooth movements. The use of attachments has not been found to improve the effectiveness of canine rotation [7].

Significant differences between the transverse widths simulated in ClinCheck® and their implementation in clinical practice have raised concerns about the predictability of treatment outcomes [8]. Zhao et al. [9] found a decrease in expansion accuracy from the first premolars to the second molars and that > 2 mm of planned increases in intermolar width significantly reduced the effectiveness of premolar expansion. All these studies were, however, based on the E30® (Align Technology) rather than the more recent SmartTrack® (Align Technology) material used with Invisalign®, and their authors did not elaborate on the clinical relevance of the discrepancies they observed.

Currently available data on the effectiveness of aligner treatment are confined almost exclusively to intramaxillary tooth movement without taking occlusion into account, even though occlusal contact adjustment does make a key difference to the success of orthodontic treatment in terms of ensuring a functional stomatognathic whole [10]. The current SmartTrack® material was introduced in 2013 to improve the predictability of aligner treatment [11]. Robertson et al. [12] have pointed out that the existing body of research into the effectiveness of aligners, and hence, the mainstay of systematic reviews on the subject is still mainly based on previous aligner materials and concepts.

The present study was designed to fill this gap in the literature as to whether, and to what extent, the transition from E30® to SmartTrack® with its different material properties may have improved the situation. We set out to answer this question by comparing the outcomes of aligner therapies performed with the new material to the outcomes simulated in the planning software, analyzing in this way the precision of implementing the transverse tooth movements and occlusal contacts thus planned.

## Materials and methods

### Patient selection

Sixty-eight patients were considered for inclusion in this retrospective study, all having received Invisalign® aligner treatment exclusively with the SmartTrack® material. Only patients with permanent dentitions were included in whom orthodontic treatment had been successfully completed. Any cases involving nonvital teeth, oral hygiene deficits, or orthodontic extraction therapy were excluded. Only 30 patients met these criteria, including 23 female patients aged 31.3 (13−50) and 7 male patients aged 25.7 (15−43) years on average at the outset of treatment.

### Models included

Four maxillary models were analyzed per patient, two reflecting the pretreatment and two the posttreatment situation: (i) a cast of the initial situation, (ii) a virtual model based on a scan of the initial situation, (iii) a clinical model (cast or intraoral scan) obtained on completion of treatment, and (iv) a model reflecting the treatment outcome as initially simulated in the planning software. STL datasets formed the basis for measurements. Baseline casts were available for all patients, were digitized with a laboratory scanner (S600 ARTI; Zirkonzahn, Irvine, CA, USA) and the STL file loaded into dental imaging software (Onyxceph® 3D Pro; Image Instruments, Chemnitz, Germany). Outcomes were available as casts for 17 patients and as intraoral scans for 13 (iTero®; Align Technology).

### Transverse parameters

**Fig. 1:**
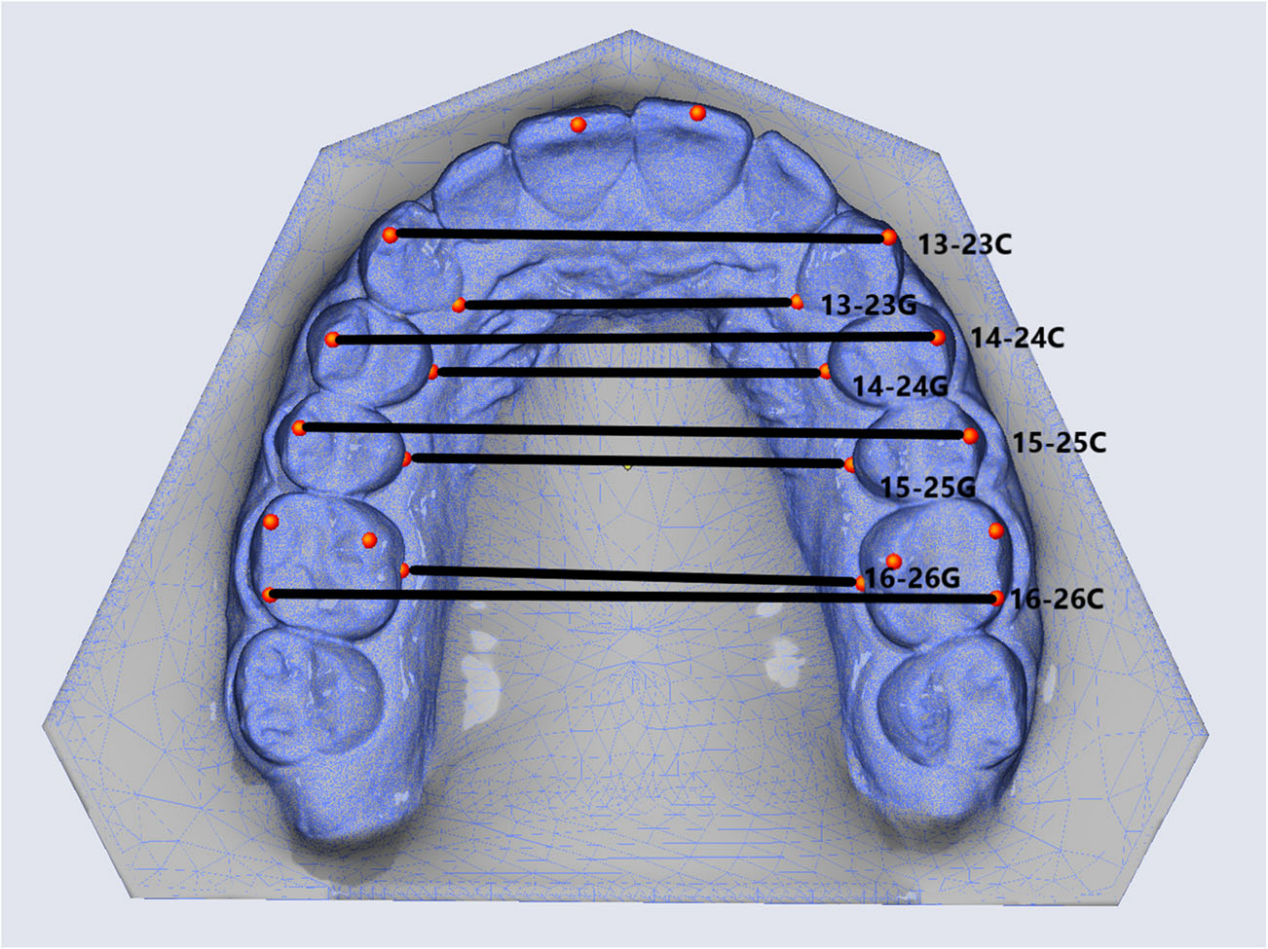
Transverse width parameters: intercanine width measured at the cusp tips (13–23C) and gingival margins (13–23G), first interpremolar width measured at the cusp tips (14–24C) and gingival margins (14–24G), second interpremolar width measured at the cusp tips (15–25C) and gingival margins (15–25G), and first intermolar width measured at the cusp tips (16–26C) and gingival margins (16–26G)

**Fig. 2:**
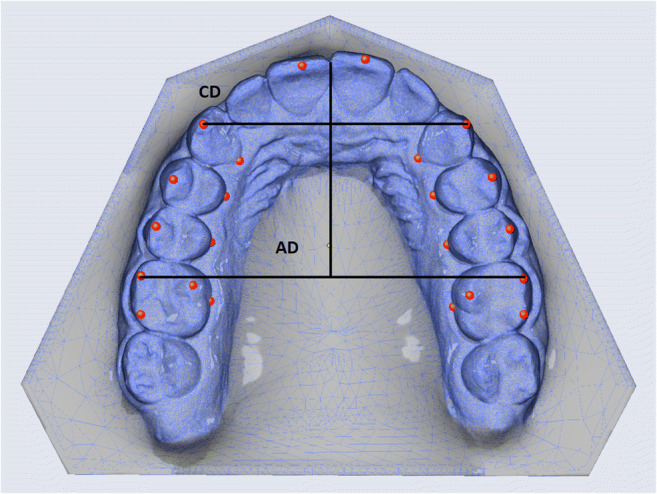
Transverse depth parameters including intercanine depth (CD) and arch/intermolar depth (AD)

**Fig. 3:**
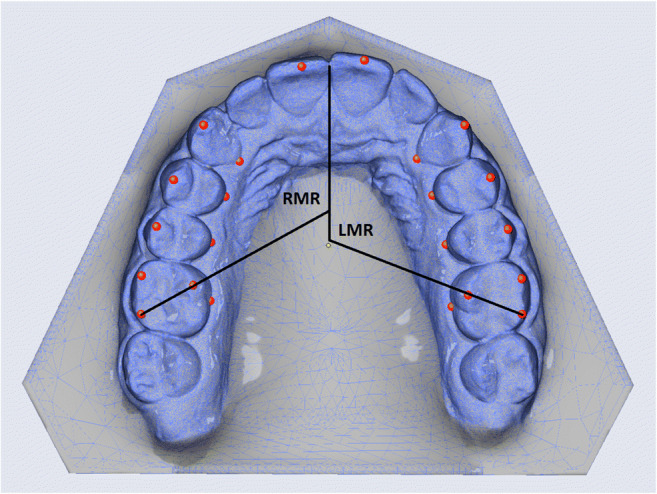
Transverse angular parameters including right and left molar rotation (RMR and LMR)

Two investigators measured the parameters also listed in Tables 1 and 2 independently, including parameters of transverse width from the canines through the first and second premolars back to the first molar, and each of these widths separately at the level of the buccal cusp tips and at the level of the palatal gingival margins (Fig. 1). Parameters of transverse depth were also measured, including intercanine depth where the line connecting both cusp tips is intersected by the midline (i.e., a perpendicular drawn from between the incisal edges of the central incisors) and intermolar depth (also referred to as “arch depth”) obtained in the same way between the mesiobuccal cusp tips of the first molars (Fig. 2). Angular parameters included the transverse rotation angles of the right and left first molars, measured between the extended line drawn through the distobuccal and mesiopalatal cusps of each first molar and the midline, and intermolar inclination measured between both of these extended lines drawn through these distobuccal and mesiopalatal cusps on both sides (Fig. 3).

**Table 1:** Summary of values measured by both investigators, expressed as quantiles (Q). The 25% quantiles (Q_1_) and 75% quantiles (Q_3_) are given to the left and right of the 50% quantiles (Q_2_), which are the median values

	Topographic sites of measurement	Investigator 1					Investigator 2						
Teeth		Simulated (planning)			Achieved (outcomes)		Simulated (planning)				Achieved (outcomes)		
Transverse width parameters (mm)		Q_1_	Q_2_	Q_3_	Q_1_	Q_2_	Q_3_	Q_1_	Q_2_	Q_3_	Q_1_	Q_2_	Q_3_
13–23	Cusp tips	0.5	0.85	1.8	0.4	0.8	1.55	0.4	0.95	1.58	0.5	1.05	1.58
13–23	Palatal gingival margins	0.3	0.7	1.65	0.28	0.9	1.38	0.3	0.8	1.45	0.38	0.8	1.8
14–24	Buccal cusp tips	1.13	2.1	2.8	0.7	1.75	3.25	1.1	1.9	2.68	1.1	1.8	2.73
14–24	Palatal gingival margins	0.75	1.7	2.43	0.8	1.05	2.43	0.88	1.7	2.73	0.7	1.1	2.33
15–25	Buccal cusp tips	1.68	2.9	3.73	1.5	2.35	3.23	1.38	2.45	3.55	1.08	2.3	3.63
15–25	Palatal gingival margins	1	1.6	2.83	0.78	1.55	2.7	1.15	1.75	2.63	0.6	1.9	2.68
16–26	Distobuccal cusp tips	0.7	1.85	2.55	0.98	1.9	2.1	0.75	1.7	2.63	1.03	1.65	2.68
16–26	Palatal gingival margins	0.5	0.95	1.5	0.58	1	1.43	0.48	0.95	1.6	0.68	1.2	1.63
Transverse depth parameters (mm)													
13/23	Cusp tips	0.3	0.75	1.8	0.2	0.6	1.6	0.38	0.9	1.6	0.28	0.6	1.4
16/26	Mesiobuccal cusp tips	0.73	1.2	1.53	0.5	0.8	1.3	0.6	1.1	1.6	0.4	1.05	1.45
Transverse angular parameters (°)													
16	Distobuccal cusp tip^a^	1.08	2.85	5.73	1.28	3	4.6	1.95	3.55	6.7	0.8	3.5	5.83
26	Distobuccal cusp tip^b^	2.08	4	6.08	2.15	3.45	6.28	2.85	4.4	8.23	1	2.5	5.93
∢16/26	Distobuccal cusp tips^c^	1.85	4.65	8.33	2.35	4.25	7.75	1.9	4.4	8.43	1.88	5.25	7.95

^a^Right molar rotation; ^b^left molar rotation; ^c^intermolar inclination. Also see Fig. 2

**Table 2:** Statistical overview of differences obtained by both investigators (I1, I2) between the pretreatment clinical versus virtual models and the posttreatment clinical models versus the virtual simulations used for planning. The median discrepancies between these latter two posttreatment model categories are also listed, as are the maximum amounts of under- and overcorrection measured for each parameter, as well as the percentages of cases in which the simulated treatment goals were achieved regardless of overcorrection and percentages indicating the efficacy of achievement

		Pretreatment clinical vs. virtual model		Posttreatment clinical vs. simulated model		Posttreatment median discrepancy clinical vs. simulated ± SD			Undercorrection (left) and overcorrection (right): maximum values		Cases (left) and efficacy (right) of simulated goals being achieved	
	Topographic sites of measurement											
Teeth		I1	I2	I1	I2	I1	I2	Δ				
Transverse width parameters (mm)		*p*	*p*	*p*	*p*	mm/°	mm/°	*p*	mm/°	mm/°	%	%
13–23	Cusp tips	.014	.190	.587	.819	0.4 ± 0.3	0.3 ± 0.2	.280	2.2	1.3	46.65	50.0
13–23	Palatal gingival margins	.117	.914	.072	.931	0.45 ± 0.3	0.35 ± 0.4	.983	2.2	3.7	28.35	78.4
14–24	Buccal cusp tips	.103	.199	.076	.837	0.5 ± 0.25	0.35 ± 0.2	.466	2.2	1.3	41.7	21.8
14–24	Palatal gingival margins	.467	.167	.225	.240	0.4 ± 0.2	0.35 ± 0.2	.845	2.0	2.2	46.65	36.2
15–25	Buccal cusp tips	.125	.176	.05	.132	0.5 ± 0.3	0.35 ± 0.4	.719	2.2	2.4	50.0	21.1
15–25	Palatal gingival margins	.044	.003	.973	.877	0.5 ± 0.45	0.35 ± 0.4	.820	2.3	3.0	56.65	31.2
16–26	Distobuccal cusp tips	.451	.550	.578	.791	0.5 ± 0.35	0.45 ± 0.2	.642	2.1	2.5	40.0	34.7
16–26	Palatal gingival margins	.420	.267	.778	.368	0.5 ± 0.3	0.55 ± 0.2	.581	2.9	2.0	50.0	48.0
Transverse depth parameters (mm)												
13/23	Cusp tips	.066	.023	.171	.017	0.3 ± 0.2	0.35 ± 0.2	.770	2.1	1.1	28.35	42.0
16/26	Mesiobuccal cusp tips	.00	.006	.017	.255	0.4 ± 0.25	0.4 ± 0.3	.090	1.9	2.2	35	38.7
Transverse angular parameters (°)												
16	Distobuccal cusp tip^a^	.299	.544	.551	.393	2.9 ± 1.9	3.2 ± 3.5	.910	14.5	7.5	30.0	96.9
26	Distobuccal cusp tip^b^	.846	.579	.750	.165	2.9 ± 2.45	3.15 ± 3.7	.750	19.0	12.4	30.0	109.4
∢16/26	Distobuccal cusp tips^c^	.011	.343	.721	.837	2.4 ± 1.65	4.6 ± 2.05	.163	19.9	6.9	36.65	83.5

^a^Right molar rotation; ^b^left molar rotation; ^c^intermolar inclination. Also see Fig. 2

### Outcome measures

Based on these transverse parameters thus measured, the pretreatment clinical casts and scanned models were compared to assess the transfer precision from cast to planning software. In the same way, the posttreatment (clinical and planning software) models were compared to assess how effectively the outcomes of expansion or contraction simulated in the planning phase had been achieved in absolute terms. In relative terms, the movements achieved (i.e., post- versus pretreatment clinical models) were expressed as percentages of the planned transverse changes (i.e., post- versus pretreatment models in the planning software) regardless of overcorrection, and percentages were also used to indicate the effectiveness of these changes.

### Occlusal contact analysis

**Fig. 4:**
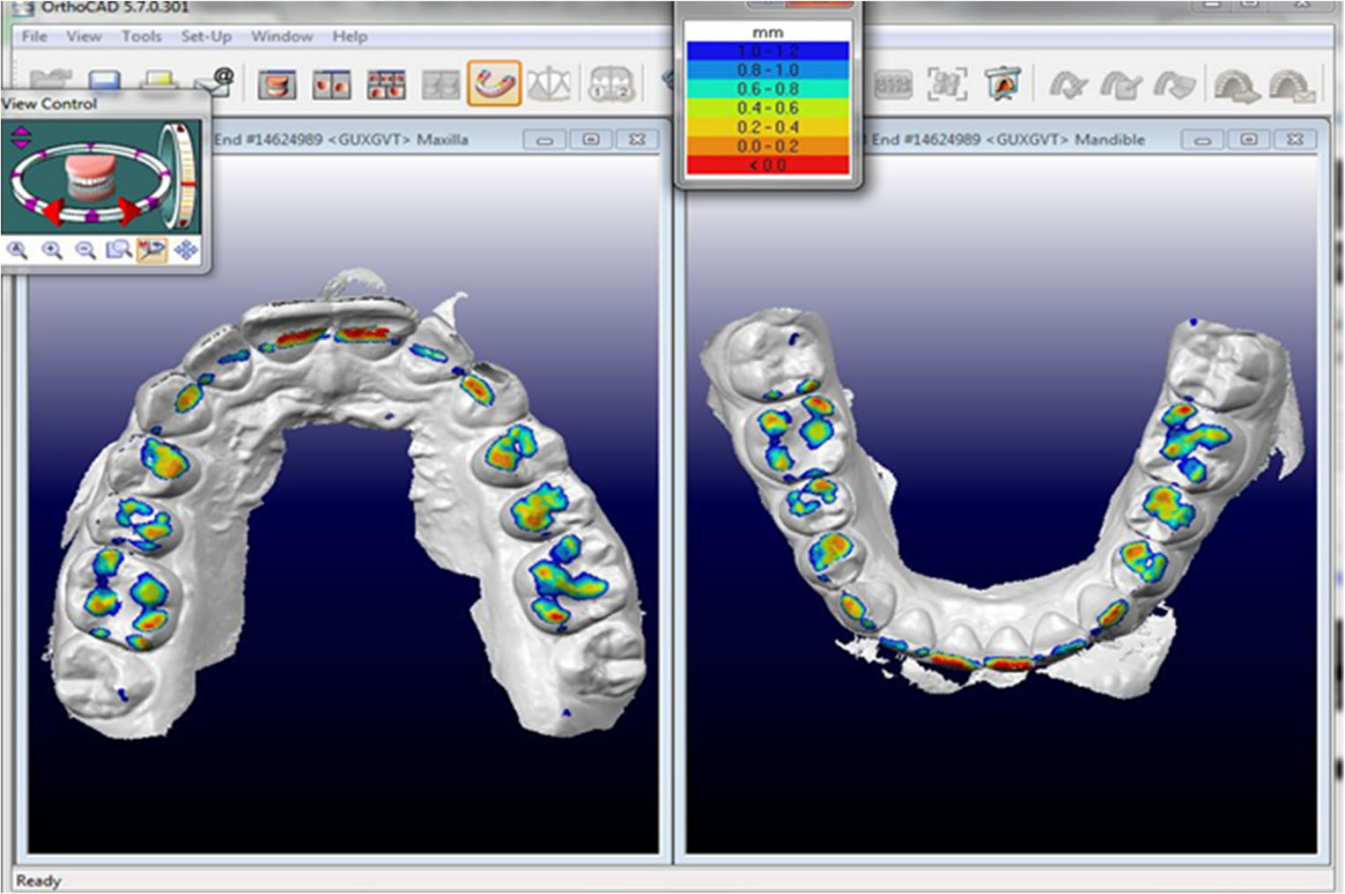
View of occlusal contacts in OrthoCAD® (Align Technology)

**Fig. 5:**
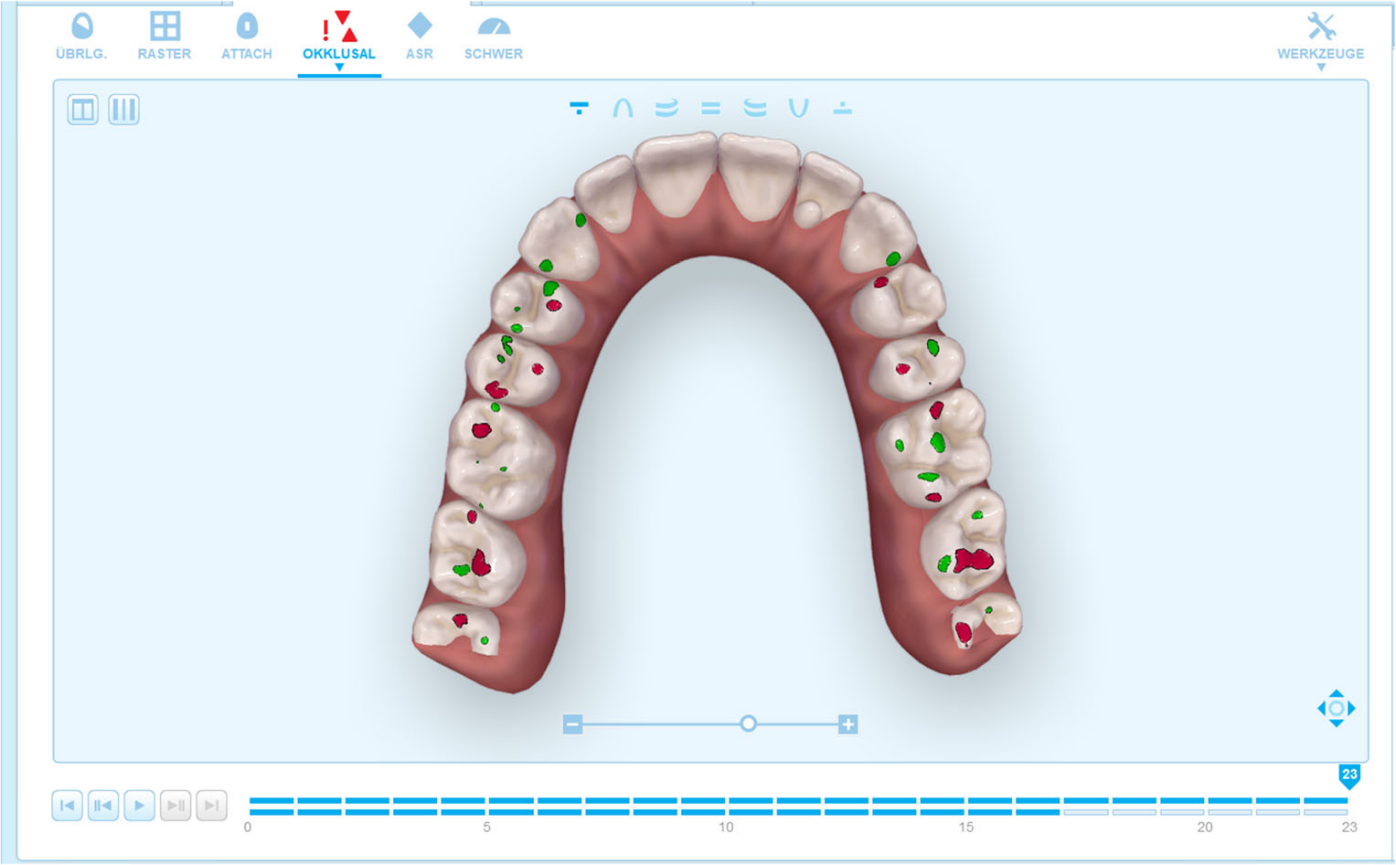
View of occlusal contacts in ClinCheck® (Align Technology)

The posttreatment models were used for this analysis. The clinical models were loaded into OrthoCAD® (Align Technology), viewed in occlusion with the contacts shown, and screenshots were taken. OrthoCAD® categorizes occlusal contacts by assigning specific colors (red, yellow, orange, green, cyan, light blue, blue) to occlusal areas of 0.0 to 1.2 mm in 0.2 mm increments (Fig. 4). The ClinCheck® planning software only uses red for intense versus green for clinically ideal contacts (Fig. 5). While red contacts in OrthoCAD® were considered equivalent to red contacts in ClinCheck®, areas ≤ 0.6 mm (yellow, orange, or green) were equated with green and will be discussed as “green” in this study. Areas > 0.6 mm, indicated by bluish colors, were disregarded as not constituting contacts.

### Statistical analysis

The sample size was calculated using IBM SPSS® Statistics 24.0 (IBM, Armonk, NY, USA). The power of the study was 90% and 5% significance level, a sample size of 30 subjects would be sufficient. The occlusal contact data were transferred to spreadsheets (Excel 2016; Microsoft, Redmond, WA, USA) and statistically analyzed using IBM SPSS® Statistics 24.0 (IBM, Armonk, NY, USA). The 13 transverse outcome parameters were evaluated for each of the four maxillary models per patient in Onyxceph®. Landmarks were manually placed, linear parameters projected to the transverse plane, the results transferred separately to a spreadsheet (Excel 2016), and statistically evaluated (IBM SPSS® Statistics 24.0). Mean values, standard deviations, and quantiles were used for descriptive statistics and Wilcoxon testing to compare the pretreatment and posttreatment models as described above. Spearman’s rank correlation coefficients were obtained to identify any significant associations between the planned (i.e., simulated) expansion of the first molars and the effectiveness of expansion achieved for each of the transverse parameters investigated. Differences were considered statistically significant at *p* < 0.05.

## Results

Table 1 lists for both investigators the interquartile ranges for their measurements of all investigated transverse linear and angular parameters as they had been simulated in the ClinCheck® planning software and as they had been clinically achieved.

### Transfer precision from casts to planning software

Table 2 summarizes the results of the Wilcoxon test applied to determine significant differences between the pretreatment clinical and virtual models as measured by both investigators. These differences reflect the precision of transferring impressions to virtual models in the ClinCheck® planning software, thus indicating the measuring accuracy of the strategy we used. The measurements of investigator 1 exhibited significant deviations for intercanine width at the cusp tips (*p* = 0.014), for second interpremolar width at the gingival margins (*p* = 0.044), as well as for intermolar depth (*p* = 0.00) and intermolar inclination (*p* = 0.011). The measurements of investigator 2 involved significant deviations for second interpremolar width at the gingival margins (*p* = 0.003), for intercanine depth (*p* = 0.023), and for intermolar depth (*p* = 0.006).

### Clinical effectiveness of transverse planning in absolute terms

Table 2 also lists the results of the Wilcoxon test applied in the same way to the differences between the posttreatment clinical and virtual models, the latter constituting the simulated treatment goals. Investigator 1 obtained significantly different results for intermolar depth (*p* = 0.017) and investigator 2 for intercanine depth (*p* = 0.017). The median values for transverse discrepancy between both posttreatment models are also listed for both investigators. The largest undercorrection compared to the simulated goals was seen for intermolar width at the gingival margins (2.9 mm) and the largest overcorrection for intercanine width at the gingival margins (3.7 mm).

### Clinical effectiveness of transverse planning in relative terms

Table 2 also shows the percentage of patients the initial goal of the planned expansion or contraction was achieved with. Using at least half of the patients as yardstick, only the parameter of second interpremolar width was seen to meet this criterion both at the cusp tips (50.0%) and at the gingival margins (56.65%), and intermolar width still achieved the desired transverse goal at the gingival margins in 50.0% of cases. Disregarding overcorrection, five of the 13 parameters did meet this criterion of ≥ 50%, including intercanine width at the cusp tips, intercanine width at the gingival margins, left molar rotation, right molar rotation, and intermolar inclination.

### Clinical effectiveness of occlusal contact planning

Table 3 provides an overview of the planned occlusal contacts and the actual clinical outcomes. Effectiveness here refers to the percentage of, for example, simulated red contacts actually resulting in clinically intense contacts (red-to-red) as opposed to how many of them turned out to be clinically well adjusted (red-to-green). The same principle applies to the simulated green contacts, including a subset materializing as clinically intense (green-to-red) and another one as clinically ideal (green-to-green). Table 3 also summarizes the overall agreement of the simulated contact patterns with the patterns seen in the clinical outcomes and how many of the simulated contacts came about at the simulated intensity (red-to-red plus green-to-green) or turned out to be ideal in the clinical outcomes regardless of the simulation (green-to-green plus red-to-green).

**Table 3:** Number of occlusal contacts and effectivenss of their materialization based on the outcomes simulated for treatment planning versus in the actual clinical outcomes. Results are expressed as quantiles; the 25% quantiles (Q_1_) and 75% quantiles (Q_3_) are given to the left and right of the 50% quantiles (Q2), which are the median values

		Contacts (*n*)			Effectiveness (%)		
		Q_1_	Q_2_	Q_3_	Q_1_	Q_2_	Q_3_
Simulated outcomes	Red	4.3	12	14	n/a	n/a	n/a
Clinical outcomes	Red → red	0	1	3.3	0	13.4	31
	Red → green	2.5	5	7	34	48.1	62.5
	Red				46	71	87.5
Simulated outcomes	Green	5	8	12	n/a	n/a	n/a
Clinical outcomes	Green → green	2	5	9	27.7	61.3	70.4
	Green → red	0	0.5	1.3	0	2.1	18.6
	Green				37.1	70	83.6
Simulated outcomes	Any contacts	15.8	18.5	23	n/a	n/a	n/a
Clinical outcomes	Any contacts	9.8	12	16.3	60.9	72.2	85
	As simulated^a^	4	6.5	10.5	22.2	41.9	61.3
	Ideal^b^	7	10	14	41.3	59.1	67.6

^a^Contacts materializing at the simulated intensity (red → red or green → green)

^b^Contacts that may be regarded as clinically “ideal” (green → green or red → green)

## Discussion

The effectiveness of transverse movement was smaller by at least 10 percentage points at the cusp tips than at the gingival margins of each tooth site (Table 2). The simulated degrees of expansion suggested an effectiveness of the clinical outcomes depending on the amount of movement, given that the mean value of expansion planned at the cusp tips exceeded the mean value planned at the gingival margins by up to 1 mm. Indeed, there was a significant negative correlation between the planned intermolar movement—at both the cusp tips and the gingival margins—and the actual movement. In other words, the greater the planned movement, the less effective it was.

Solano et al. [8], in their analysis of posttreatment virtual models (ClinCheck®) versus clinical models to determine the effectiveness of expansion, found a lack of precision (*p* < 0.05) for intercanine depth and for all width measurements (canines, first and second premolars, first molars) both at the cusp tips and at the palatal margins.

Of the posttreatment clinical models in the present study, 17 were obtained with a laboratory scanner (S600 ARTI; Zirkonzahn) that is adequately effective for clinical use, given a documented trueness of 65.9 ± 1.33 μm and precision of 20.7 ± 4.34 μm for entire-jaw scans [13]. For the other 13 virtual models, an intraoral scanner (iTero®; Align Technology) with a documented trueness of 9.8 ± 2.5 μm and precision of 7.0 ± 1.4 μm was used for this purpose [14]. These accuracy values do not suggest that the digitization technique may have caused the differences in intercanine and intermolar depth we observed between the posttreatment clinical and the simulation models.

Although the statistical precision of achieving the planned movements was low in the present study, it should be considered that deviations under 1 mm are not clinically relevant. What does matter in clinical practice is whether the planned tooth movement (expansion or contraction) has been achieved completely, regardless of the presence or absence of overcorrection. Looking at the percentage values for the various tooth sites in Table 2, the planned expansion or contraction was achieved in 45% of cases. Decisions whether to allow for overcorrection in the planning stage need to be made on a case-by-case basis depending on the goal of treatment. There is also a need to keep reevaluating the cases over the course of treatment to detect and address any transverse movements that may not proceed according to plan at an early stage.

Another goal was to verify the measuring method used in the present study by having two investigators perform the measurements. The median discrepancies which both of them incurred between the posttreatment clinical and planning models differed by up to 0.15 mm or 2.2° (see Table 2) based on the highest median measurements of 2.35 mm or 5.25° (see Table 1). There were no indications for a significant difference between both investigators in the results concerning effectiveness. Our comparison of both datasets does suggest that Onyxceph® is a serviceable environment to measure the parameters and calculate the effectiveness of transverse tooth movement and discrepancies involved.

The extent of occlusal contacts materializing as initially planned was reviewed by an investigator based on an anatomical classification of occlusal surfaces introduced by Delong et al. [15]. Image distortion due to different software applications precluded a computer-based analysis in the form of superposing screenshots of the posttreatment clinical and planning models [16]. ClinCheck® simulates the occlusal treatment goal in the form of green and red contacts (Fig. 4). Green contacts (summarized as red-to-green and green-to-green in Table 3) are key to a successful treatment outcome. Red contacts, by contrast, may materialize as intense or even premature contacts, which is why many clinicians routinely eliminate all red contacts during planning.

Our findings show, however, that red contacts materialize far less than green contacts, given an effectiveness of 13.4% versus 48.1%. Indeed, an initial median of 12 red contacts resulted in just one red and five green clinical contacts (Table 3). While 61.3% of green contacts materialized, only 2.1% of red contacts did (see Table 3). *Any* of the simulated contacts (red-to-red, red-to-green, green-to-green, green-to-red) were found to materialize in 72% and clinically ideal ones (red-to-green, green-to-green) in 59% of all instances. Given both of these approximately 60% rates of ideal and green-to-green contacts, eliminating the red contacts from the initial simulation does not make a difference. In other words, 40% of the simulated contacts will never materialize, and achieving about 60% of simulated contacts is adequate to ensure a clinically favorable contact pattern.

Spearman’s rank correlation coefficient was obtained, confirming a significant positive correlation (rho = 0.497) of green contacts in ClinCheck® with effective green-to-green contacts. In other words, the more red contacts are simulated in ClinCheck®, the more red-to-green contacts will develop clinically, which reaffirms that there is no need to remove each and every red contact in the ClinCheck® planning, as many of these red contacts will, in clinical reality, end up as ideal contacts by the end of treatment. The occlusion should, however, be checked not only immediately upon completion of treatment, but allowances should also be made for settling. Any overcorrections in the planning stage [17] can distort the effectiveness and precision of the clinical outcome, even though most of these deviations will be minor and clinically not relevant.

## Limitations

The relatively small amounts of simulated expansion—given a maximum of 5.2 mm—may have increased the risk of error in placing reference points.

## Conclusions

• Invisalign® therapies with SmartTrack® aligners involved a 45% effectiveness in achieving treatment objectives of transverse contraction or expansion. It is therefore possible to include overcorrections in the planning stage.

• The effectiveness of achieving transverse values as planned was generally not increased with SmartTrack® compared to the previously used Ex30® material.

• Pretreatment by rapid maxillary expansion should be considered in some patients presenting large transverse discrepancies between the maxilla and mandible.

• Overcorrections are also an option in simulating occlusal contacts during the planning stage, given an effectiveness of 59.1% in achieving clinically ideal contacts.

• Any overcorrection in the planning stage will always, if to a minor or even irrelevant degree, reduce the precision of achieving the clinical outcome as simulated.

• Statistically significant discrepancies were observed between the simulated and the clinical outcomes of Invisalign® treatment with SmartTrack® aligners.
